# Sage Insights Into the Phylogeny of *Salvia*: Dealing With Sources of Discordance Within and Across Genomes

**DOI:** 10.3389/fpls.2021.767478

**Published:** 2021-11-24

**Authors:** Jeffrey P. Rose, Ricardo Kriebel, Larissa Kahan, Alexa DiNicola, Jesús G. González-Gallegos, Ferhat Celep, Emily M. Lemmon, Alan R. Lemmon, Kenneth J. Sytsma, Bryan T. Drew

**Affiliations:** ^1^Department of Biology, University of Nebraska at Kearney, Kearney, NE, United States; ^2^Department of Botany, University of Wisconsin–Madison, Madison, WI, United States; ^3^CONACYT, Instituto Politeìcnico Nacional, CIIDIR – Durango, Durango, Mexico; ^4^Department of Biology, Faculty of Arts and Sciences, Kırıkkale University, Yahşihan, Turkey; ^5^Department of Biological Science, Florida State University, Tallahassee, FL, United States; ^6^Department of Scientific Computing, Florida State University, Tallahassee, FL, United States

**Keywords:** anchored hybrid enrichment, cyto-nuclear discordance, distance metrics, incongruence, Lamiaceae, Robinson–Foulds distance, *Salvia*

## Abstract

Next-generation sequencing technologies have facilitated new phylogenomic approaches to help clarify previously intractable relationships while simultaneously highlighting the pervasive nature of incongruence within and among genomes that can complicate definitive taxonomic conclusions. *Salvia* L., with ∼1,000 species, makes up nearly 15% of the species diversity in the mint family and has attracted great interest from biologists across subdisciplines. Despite the great progress that has been achieved in discerning the placement of *Salvia* within Lamiaceae and in clarifying its infrageneric relationships through plastid, nuclear ribosomal, and nuclear single-copy genes, the incomplete resolution has left open major questions regarding the phylogenetic relationships among and within the subgenera, as well as to what extent the infrageneric relationships differ across genomes. We expanded a previously published anchored hybrid enrichment dataset of 35 exemplars of *Salvia* to 179 terminals. We also reconstructed nearly complete plastomes for these samples from off-target reads. We used these data to examine the concordance and discordance among the nuclear loci and between the nuclear and plastid genomes in detail, elucidating both broad-scale and species-level relationships within *Salvia*. We found that despite the widespread gene tree discordance, nuclear phylogenies reconstructed using concatenated, coalescent, and network-based approaches recover a common backbone topology. Moreover, all subgenera, except for *Audibertia*, are strongly supported as monophyletic in all analyses. The plastome genealogy is largely resolved and is congruent with the nuclear backbone. However, multiple analyses suggest that incomplete lineage sorting does not fully explain the gene tree discordance. Instead, horizontal gene flow has been important in both the deep and more recent history of *Salvia*. Our results provide a robust species tree of *Salvia* across phylogenetic scales and genomes. Future comparative analyses in the genus will need to account for the impacts of hybridization/introgression and incomplete lineage sorting in topology and divergence time estimation.

## Introduction

It has long been recognized that when generating multilocus nucleotide sequence data, different datasets may generate alternative gene tree topologies and, by extension, differing hypotheses of relationships among species (Pamilo and Nei, 1988; Rieseberg and Soltis, 1991; Maddison, 1997). The underlying causes for why such differing gene tree topologies may exist (apart from analytical artifacts) have been well-discussed in the literature, and include gene duplication, incomplete lineage sorting (ILS), lateral gene transfer, and introgression/hybridization (Degnan, 2018). These processes are not mutually exclusive, and the history of one locus may be shaped by multiple processes. For many years, the solution to deal with such discordance was to analyze incongruent datasets separately, attempt to reconcile these topologies into a consensus tree, or concatenate all loci together to generate a “total-evidence” hypothesis of the species relationships (Ané et al., 2007). The concept of genomic concordance, coupled with new methods for estimating species trees while taking into account ILS and/or horizontal gene flow have been important advances in the field of systematic biology (Ané et al., 2007; Baum, 2007; Heled and Drummond, 2009; Mirarab et al., 2014; Yu and Nakhleh, 2015; Edwards et al., 2016; Solís-Lemus and Ané, 2016).

Contemporaneous with these computational advances, new sequencing technologies have facilitated relatively easy and cost-effective sequencing of complete organellar genomes and hundreds to thousands of nuclear loci. The confluence of these two areas of biology has made for an exciting time for studies in systematic biology but has also presented challenges as to how to best analyze these datasets. For example, individual loci may have relatively little phylogenetic information and thus, confound analyses that rely on individual gene trees. In addition, the computational ability of many current algorithms are challenged by the number of terminals present in the species tree and especially the phylogenetic network that a researcher wishes to estimate (Hejase and Liu, 2016; Solís-Lemus and Ané, 2016; Rose et al., 2021). Despite these challenges, an ever-increasing proportion of phylogenomic studies employ methods that account for sources of intra-genomic discordance, especially due to ILS. While methods that employ the multispecies coalescent only are relatively fast and tractable on datasets with dozens to hundreds of terminals, it is increasingly clear that hybridization and introgression are important processes at both shallow and deep phylogenetic scales, and this affects all branches of the Tree of Life (Folk et al., 2018). If horizontal gene flow has been operative, the species tree estimated by methods that only account for ILS may differ substantially from the “true” species tree not only topologically, but also in branch lengths (Leaché et al., 2014). The misestimation of both properties may impact myriad downstream analyses.

Apart from discordance among nuclear loci, gene trees may differ among genomes. This phenomenon is well-known and often referred to as “cytonuclear discordance” (Rieseberg and Soltis, 1991). In plants, this is best demonstrated in cases of putative “chloroplast capture” which have been documented for decades (e.g., Smith and Sytsma, 1990). Such discordance has generally been taken as evidence of horizontal gene flow, even though organellar genomes are also susceptible to ILS, albeit with a much faster expected time to coalescence, relative to nuclear loci. Simulation studies have generally confirmed that most cases of chloroplast (technically plastid) capture are indeed best explained by horizontal gene flow, rather than ILS (Folk et al., 2017; Morales-Briones et al., 2018; Rose et al., 2021).

Therefore, a better understanding of the evolutionary history of clades requires an assessment of the contribution of each of the multiple processes responsible for the discordance among loci. This assessment is important not only for producing a robust phylogenetic hypothesis, but also for selecting methods, taxa, and loci appropriate for the downstream analyses of trait evolution, historical biogeography, and diversification rates. Robust phylogenetic hypotheses are also crucial for making informed decisions to ensure an accurate and stable taxonomic circumscription, from the species level to higher-level classifications.

Sage and its relatives (*Salvia* L.) comprise ∼1,000 species, with a subcosmopolitan distribution across a diversity of habitat types (Kriebel et al., 2019). It is the largest genus within the mint family (Lamiaceae) and one of the largest genera of plants. There are three broadly defined centers of diversity of *Salvia* (Walker et al., 2004): East Asia (∼100 spp.; subgenus (subg.) *Glutinaria*; Hu et al., 2018), the Mediterranean (∼250 spp.; subg. *Salvia*, *Sclarea*), and especially, Mexico, Central, and South America (∼580 spp., subg. *Calosphace*; González-Gallegos et al., 2020). *Salvia* is not only of interest from an economic perspective, given its culinary use (e.g., chia: *S. hispanica* L.; rosemary: *S. rosmarinus* (L.) Spenn.; sage: *S. officinalis* L.), but also in its horticultural importance (e.g., blue sage: *S. nemorosa* L.; pineapple sage: *S. elegans* Vahl; Russian sage*: S. yangii* B.T.Drew).

*Salvia* is florally diverse (Kriebel et al., 2019, 2020) and easily characterized by the presence of two stamens with an elongate (or swollen) anther connective, in addition to several micromorphological synapomorphies (Drew et al., 2017). In many species of *Salvia*, the connective has been variously modified – possibly multiple times – into a staminal lever mechanism to facilitate effective pollination (Claßen-Bockhoff et al., 2003, 2004; Walker and Sytsma, 2007; Wester and Claßen-Bockhoff, 2007; Celep et al., 2020).

As a result of its practical importance to humans, distribution, size and taxonomic complexity, and unique pollination biology, *Salvia* has received considerable attention from systematists and pollination biologists. Early in the study of the phylogenetic placement of *Salvia*, it was realized that the genus was polyphyletic or broadly paraphyletic, with several smaller genera embedded within it (Walker and Sytsma, 2007). Subsequent phylogenetic analyses have confirmed that five previously recognized small genera (*Dorystaechas* Boiss. & Heldr., *Meriandra* Benth., *Perovskia* Kar., *Rosmarinus* L., and *Zhumeria* Rech.f. & Wendelbo) are nested within several clades of *Salvia* (Walker and Sytsma, 2007; Drew and Sytsma, 2012; Will and Claßen-Bockhoff, 2014, 2017; Drew et al., 2017). To accommodate these small genera, Drew et al. (2017) and Kriebel et al. (2019) presented an expanded concept of *Salvia*, recognizing a total of 11 subgenera, although their informal circumscription of subg. “*Heterosphace*” represents a geographically diverse assemblage of lineages.

To date, most phylogenetic studies of *Salvia* have relied on plastid or nuclear ribosomal external transcribed spacer (ETS) and especially internal transcribed spacer (ITS) sequences (Walker and Sytsma, 2007; Jenks et al., 2013; Will and Claßen-Bockhoff, 2014, 2017; Dizkirici et al., 2015; Walker et al., 2015; Fragoso-Martínez et al., 2018; Hu et al., 2018). The resolution at multiple phylogenetic scales with these markers is variable by clade and, while there has been evidence for several deeper-level clades along the backbone of *Salvia*, relationships among them have usually either not been resolved or well supported. Discordance among plastid and nuclear ribosomal loci is generally found but not well-discussed or quantified (but see Walker et al., 2015). In cases where relationships differ across studies and marker sets, it is not clear if the differences are due to a true discordance in genealogical history or are from errors in the phylogenetic estimation (cf. Will and Claßen-Bockhoff, 2017: Figure 1). Drew et al. (2017) further investigated the backbone relationships in *Salvia* using two low-copy nuclear loci. While several key nodes remained unresolved and there was a clear conflict between the loci, they found an increased resolution for the backbone relationships. More recently, Zhao et al. (2020) used complete plastomes from seven of 11 subgenera and recovered a nearly fully resolved backbone across *Salvia* except for uncertainty in the placements of subg. *Perovskia* and *Rosmarinus*.

Multilocus nuclear datasets from anchored hybrid enrichment (AHE) have been successfully used to resolve deep and shallow level relationships across multiple angiosperm lineages, including *Salvia* (Fragoso-Martínez et al., 2017; Kriebel et al., 2019). Previously, we presented a species tree of 35 *Salvia* exemplars from 10 of 11 subgenera based on 316 nuclear loci using concatenation and one coalescent method (Kriebel et al., 2019). While this topology is congruent with the plastome phylogeny of Zhao et al. (2020) in areas where the two studies overlap in subgeneric sampling, several factors bear further consideration in Kriebel et al. (2019). First, the branching order of subg. “*Heterosphace*”, *Salvia*, and *Sclarea* is not fully supported. Second, the monophyly of subg. *Audibertia* is not fully supported. Third, within subg. *Calosphace*, section (sect.) *Axillares* was recovered as a sister to the “*Hastatae* clade”. instead of sister to all other *Calosphace*, in conflict with several previous studies (Jenks et al., 2013; Drew et al., 2017; Fragoso-Martínez et al., 2018). The placement of sect. *Axillares* has important implications for understanding character evolution in subg. *Calosphace* (e.g., Fragoso-Martínez et al., 2018; Kriebel et al., 2019, 2020, 2021).

Given the relatively sparse sampling of *Salvia* diversity from previous phylogenomic analyses, as well as the limited exploration of any discordance surrounding the backbone relationships in *Salvia*, we aimed to sample the species diversity in *Salvia* better using AHE to fulfill several goals. (1) Fully resolve the backbone of *Salvia* and assess the monophyly of the subgenera, quantifying discordance and accounting for both ILS and horizontal gene flow. (2) Generate a species tree for a much broader species-level sampling of *Salvia*, testing the efficacy of the AHE data for resolving shallow-level relationships. (3) Examine cytonuclear discordance at multiple phylogenetic scales by mining off-target organellar reads.

## Materials and Methods

### Species Sampling in *Salvia* and Outgroups

In total, the analyses consisted of 190 samples, including 179 *Salvia* and all subgenera recognized by Kriebel et al. (2019) except for the small subg. *Meriandra* (Benth.) J.B.Walker, B.T.Drew & J.G.Gonz lez. For ease of discussion, we will refer to the “*Heterosphace*” clade as a subgenus, although we acknowledge that this is not a formally named taxon. Within the subtribe Salviinae, we sampled the clade sister to *Salvia* (six species of *Lepechinia* and one species of *Melissa*: Drew and Sytsma, 2011, 2012, 2013). The ultimate outgroup consisted of samples from all the remaining subtribes of Mentheae, which represents a monophyletic group (Drew and Sytsma, 2012): Lycopinae (*Lycopus uniflorus* Michx.), Menthinae (*Clinopodium mexicanum* (Benth.) Govaerts), Nepetinae (*Agastache pallida* (Lindl.) Cory), and Prunellinae (*Prunella vulgaris* L.).

### Anchored Hybrid Enrichment: Library Preparation, Enrichment, Sequencing, and Nuclear Locus Assembly

Total DNA was extracted from the silica gel-dried or fresh leaf tissue using a DNeasy Plant Mini Kit (Qiagen, Valencia, CA, United States). The DNA concentrations were verified using a Qubit^®^ 2.0 Fluorometer (Life Technologies, Eugene, OR, United States). We used the AHE method (Lemmon and Lemmon, 2012). As the samples were sequenced across several years of study, we enriched them using slightly different probe sets (Supplementary Data Sheet S1). The samples sequenced early on in our studies utilized a generic angiosperm kit that targets 517 loci (Buddenhagen et al., 2016; Mitchell et al., 2017) or the *Salvia*-specific probes utilized in Kriebel et al. (2019), designed using the genome skimming of several *Salvia* species. The *Salvia*-specific probes targeted the same regions as the generic angiosperm kit, only neighboring exons could be combined and target regions extended, resulting in a target of 291 moderately conserved, low copy nuclear loci and their variable flanks. The library preparation, enrichment, assembly, and alignment of nuclear loci were performed at the Florida State University Center for Anchored Phylogenomics^1^ and are described in detail in Kriebel et al. (2019). Because of the large number of samples, a substantial number of loci were lost during the orthology assessment, resulting in 123 recovered loci. Nine additional loci were lost during trimming and masking due to excessive missing data, resulting in a final dataset of 114 loci.

### Nuclear Dataset 1: Complete Dataset

To examine the monophyly of the subgenera (when represented by multiple samples) and assess shallow-level relationships, we assembled species trees using all accessions. First, we concatenated all loci and generated a maximum likelihood species tree in RAxML v.8.2.11 (Stamatakis, 2014) under GTR + Γ. We assessed the branch support with 500 rapid bootstrap (BS) replicates.

Second, we generated a species tree under the multispecies coalescent using ASTRAL-III (Zhang et al., 2018). Using a batch Perl script, we generated individual gene trees using RAxML under GTR + Γ, assessing the branch support for each locus with 100 rapid BS replicates. We analyzed all the maximum likelihood gene trees in ASTRAL, measuring the branch support in two ways: by using the RAxML BS trees as input to ASTRAL with 100 replicates, and also by calculating the ASTRAL local posterior probability (LPP) (Sayyari and Mirarab, 2016) for each quadripartition.

To detail the gene tree conflict/support for each clade in the species tree, we used Phyparts (Smith et al., 2015). Phyparts takes an estimate of a species tree and set of rooted gene trees and provides four numbers for each clade: the number of loci supporting a clade, the number of loci supporting the main conflicting clade, the number of loci supporting all other conflicting clades, and the number of loci without information for a relationship. Trees were optimally rooted with our outgroups outside of Salviinae, but in cases where these were missing, we rooted trees with *Melissa* and/or *Lepechinia*. Gene trees that contained only *Salvia* were excluded from the Phyparts analysis. Note that rooting with Salviinae may inflate the gene tree support for the monophyly of *Salvia* and possibly also show misleading support or conflict for the relationships among *Lepechinia*, *Melissa*, and *Salvia*. However, we allowed this potentially incorrect rooting because our chief interest was in the relationships within *Salvia*. To mitigate the effects of uncertainty in the gene tree estimation providing artificial conflict (or support) for clades, we collapsed the branches in each gene tree with <33% BS.

### Nuclear Dataset 1: Gene Tree Distances

We further examined the patterns of gene tree discordance to test if the observed gene tree discordance across *Salvia* and its constituent subgenera are consistent with the expectation under ILS alone. To do this, we first generated 1,000 gene trees under the multispecies coalescent using the *treesim.contained_coalescent* function in DendroPy v.4.5.2 (Sukumaran and Holder, 2010) using the ASTRAL species tree as the “true” tree. To compare the observed discordance with what would be expected under ILS, we measured the pairwise tree distance of each gene tree (expected and observed) from the ASTRAL species tree using three metrics, considering branching order alone and ignoring branch lengths: the Robinson–Foulds distance (RF) (Robinson and Foulds, 1981), the method proposed by Nye et al. (2006), and the clustering information (CI) metric proposed by Smith (2020a). Calculations were made on complete gene trees or gene trees pruned to the subgenus of interest, as appropriate. Since some of the observed gene trees were missing terminals, we only used observed gene trees which contained >75% of all terminals in the clade of interest. Gene tree distances were calculated using the “TreeDist” package in R (Smith, 2020b), collapsing all the branches in the observed gene trees with <33% BS. The distances were normalized so that they ranged from 0 to 1, with 0 indicating complete agreement between the gene tree and species tree. We tested for mean differences observed in the gene tree discordance among clades using a one-way ANOVA with *post hoc* testing using the Tukey Test with the *aov* and *glht* functions in the “stats” and “multcomp” (Hothorn et al., 2008) R packages, respectively. We tested for differences in the mean gene tree discordance between observed and expected gene trees using a two-tailed Welch’s *t*-test.

### Nuclear Dataset 2: Placeholder Dataset

Our second nuclear dataset investigated deeper phylogenetic relationships in *Salvia*, accounting for both ILS and horizontal gene flow. Because the existing methods for inferring phylogenetic networks are computationally demanding for datasets with more than several dozen terminals, we constructed a dataset of one representative for each subgenus. For each subgenus placeholder, we selected the sample with the greatest number of captured loci, and in the case of ties, the total number of aligned bp. We did not allow any missing data, yielding a matrix of 57 loci for 10 species of *Salvia* plus *Lepechinia chamaedryoides* (Balb.) Epling as the outgroup. To reconstruct the phylogenetic networks, we first generated concordance factors for each possible quartet. Using a batch script, we ran MrBayes v.3.2.6 (Ronquist et al., 2012) to find the best gene tree for each locus. The gene trees were inferred under GTR + I + Γ using three runs of three chains each for five million generations each with sampling every 5,000 generations with a chain temperature of 0.4, swap frequency of 500 generations, and a 30% burnin. Following the MrBayes analysis, a Bayesian concordance analysis on the posterior sample of gene trees was conducted in BUCKy v.1.4.4 (Ané et al., 2007; Larget et al., 2010) with 100,000 post-burnin generations and the amount of *a piori* discordance among loci set to the default of 1. This analysis calculates all possible quartets and prunes on the MrBayes gene trees to all but the four terminals of interest. Then, BUCKy is run on each pruned gene tree to generate a table of all quartet concordance factors (CFs) and their SEs. Using these CFs, we generated a preliminary population tree using Quartet MaxCut (Snir and Rao, 2012).

Using the BUCKy CFs and the Quartet MaxCut tree, we calculated a phylogenetic network with the SNaQ function in the Julia package PhyloNetworks (Solís-Lemus and Ané, 2016; Solís-Lemus et al., 2017). This package uses maximum pseudo-likelihood to fit a network while also accounting for ILS. PhyloNetworks considers quartet topologies only and does not take into account information from branch lengths in individual gene trees. Furthermore, PhyloNetworks assumes a level-1 network: a network where each hybrid node only has one lineage transferring genetic material horizontally. We first tested the fit of models allowing from 0–5 reticulation events (h) and compared the models using their pseudo-likelihood score. The best network model was selected by examining at which value of h the pseudo-likelihood score plateaus, following the recommendation of Solís-Lemus et al. (2017). For each value of h, we selected the best network over 30 search replicates. We examined the branch support on the best phylogenetic network using the bootsnaq function with 50 runs of 10 replicates each.

### Plastome Assembly and Phylogenetic Analysis

We assembled the nearly complete plastomes of the Salviinae samples by mapping the off-target reads to previously published plastomes of *Salvia* for the ingroup or *Melissa* for the outgroup Salviinae. The assembly of the plastomes was conducted in Geneious v.10.2.3 (Kearse et al., 2012), following the procedure of Rose et al. (2021). For outgroup Salviinae, we used the whole plastome sequence of *Melissa yunnanensis* C.Y.Wu & Y.C.Huang (GenBank accession MT634148.1) as a reference. For *Salvia*, we constructed a “super” reference sequence based on the strict consensus of 18 GenBank plastomes (Supplementary Data Sheet S2) aligned with MAFFT v.7.023b (Katoh and Standley, 2013) under default parameters.

We used Geneious to map all the forward and reverse reads from our sequences by first trimming all raw reads, and then assembling them to the appropriate reference using an iterative refinement of up to five times with the default Geneious mapper and medium sensitivity. Consensus sequences were generated using the strict consensus approach. If the coverage for a particular site was <7, the consensus nucleotide was scored as a gap. Unmapped regions were treated as missing data and reads mapped to multiple positions were excluded from consensus calculations. Newly generated plastomes were aligned with the aforementioned GenBank sequences using MAFFT with default parameters. Ambiguously aligned regions were removed manually and were generally distinguished by putative inversions, repeat regions, an abundance of gaps, and/or uncertain base calls.

A plastome tree was inferred in RAxML under GTR + Γ with 500 rapid BS replicates. As described above in Section “Nuclear Dataset 1: Gene Tree Distances”, we measured the tree-to-tree distances between the entire plastome tree and its subclades to the ASTRAL species tree.

## Results

### Dataset Metrics

The aligned locus length for the 114 loci ranged from 105–3,671 bp, with a mean length of 1,133 bp. The samples contained sequence data for an average of 96.25 loci, with most locus dropout in the non-Salviinae outgroups. We were able to extract the majority of the plastome, with aligned plastomes totaling 157,683 bp.

### Subgeneric Monophyly and Major Relationships in *Salvia*

**FIGURE 1:**
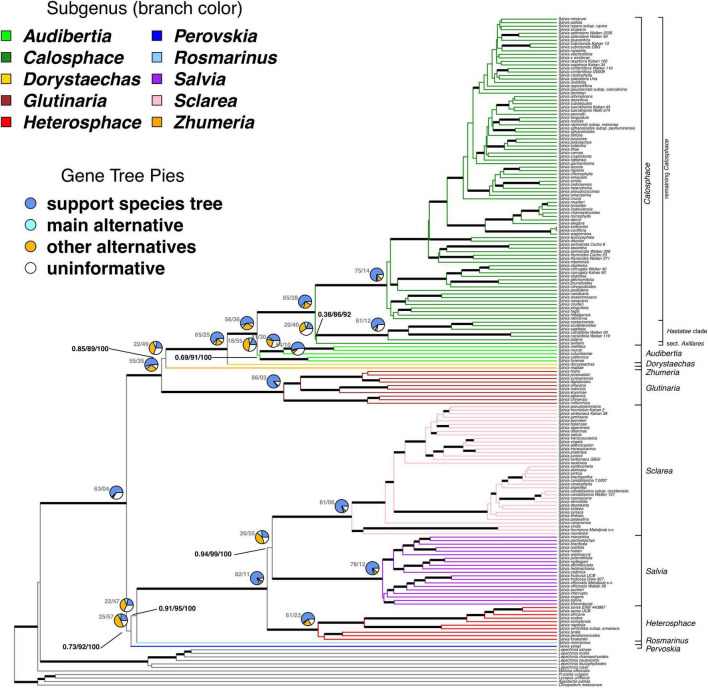
The ASTRAL species tree of *Salvia* and outgroups. The ingroup branches are colored by subgenus and the subgenera are also labeled to the right. The major clades as discussed in the text are also indicated for subg. *Calosphace*. Thickened branches denote those with >0.95 ASTRAL local posterior probability. Pies at major nodes summarize the percentage of various phylogenetic signals across 101 gene trees which can be rooted. The numbers at the left of the pies show the total number of gene trees in which the clade is found, followed by the total number of gene trees that conflict with that clade. The remainder of the gene trees, if any, do not provide information on that particular relationship. The numbers at selected, incompletely supported nodes show the ASTRAL local posterior probability followed by the ASTRAL bootstrap support and the bootstrap support from the concatenated maximum likelihood analysis, summarized on the ASTRAL species tree. For support across all branches, see Supplementary Figures S1, S2, S4. For support on the best-scoring maximum likelihood tree, see Supplementary Figure S3.

We were able to root 101 of the 114 gene trees. Species trees resulting from concatenation and accounting for ILS with ASTRAL are completely congruent in the major backbone relationships in *Salvia*, although support for these relationships sometimes varies across the approach and support metrics (Figure 1 and Supplementary Figures S1–S4). The ASTRAL normalized quartet score, or proportion of the gene tree quartet trees satisfied by the species tree, is 0.91, suggesting a clear underlying topology despite some discordance. The monophyly of *Salvia* is strongly supported by all measures of support (ASTRAL LPP = 1.0/ASTRAL BS = 100/concatenated BS = 100). In addition, the monophyly of each subgenus for which we had multiple samples is strongly supported by BS/LPP and by the vast majority of loci, with two exceptions. First, subg. *Heterosphace*, while unambiguously supported by measures of statistical support (ASTRAL LPP = 1.0/ASTRAL BS = 100/concatenated BS = 100), has 23/84 (27%) informative loci conflicting its monophyly. Second and more strikingly, the monophyly of subg. *Audibertia* is poorly supported by ASTRAL LPP (0.69) with more loci conflicting its monophyly than supporting it (55/73, 75%). However, its monophyly is more strongly supported by the other metrics (ASTRAL BS = 91/concatenated BS > 99), although both sections of subg. *Audibertia*: sects. *Audibertia* (*S. columbariae* Benth., *S. mellifera* Greene, *S. munzii* Epling) and *Echinosphace* (*S. californica* Brandegee, *S. funerea* M.E. Jones) are more strongly supported as monophyletic.

The earliest divergence in *Salvia* involves two major clades. First is a clade formed by the most recent common ancestor (MRCA) of subg. *Glutinaria* and *Calosphace* (ASTRAL LPP = 1.0/ASTRAL BS = 100/concatenated BS = 100). Subgenus *Glutinaria* is sister to all remaining subgenera, with a grade formed by the successive sisters of subg. *Zhumeria* and *Dorystaechas*, and subg. *Audibertia*, sister to subg. *Calosphace*. All of these relationships are strongly supported with the exception of the placement of subg. *Glutinaria* (ASTRAL LPP = 0.85/ASTRAL BS = 89/concatenated BS > 99; 22/71 informative gene trees).

The second major clade is formed by the MRCA of subg. *Perovskia* and *Salvia*. The monophyly of this clade is not fully supported (ASTRAL LPP = 0.73/ASTRAL BS = 92/concatenated BS = 100; 25/82 informative gene trees), nor are many of the intersubgeneric relationships within it (Figure 1). Subgenus *Perovskia* is sister to subg. *Rosmarinus* + *Heterosphace* + *Salvia* + *Sclarea* (ASTRAL LPP = 0.91/ASTRAL BS = 95/concatenated BS = 100; 22/69 informative gene trees). While the monophyly of subg. *Heterosphace* + *Salvia* + *Sclarea* is fully supported, relationships among the subgenera are slightly less certain, with subg. *Salvia* sister to *Sclarea* being the best resolution of relationships (ASTRAL LPP = 0.94/ASTRAL BS = 99/concatenated BS = 100; 26/81 informative gene trees).

The backbone of the plastome tree is nearly identical to that of the nuclear species trees (Supplementary Figure S5), with all major nodes and monophyly of the subgenera receiving maximal support except for the placement of subg. *Glutinaria* (BS = 89). The only major topological difference is that subg. *Perovskia* is weakly supported as sister to *Rosmarinus* (BS = 47).

### Infrageneric Relationships, Shallow-Scale Resolution, and Gene Tree Discordance

Within subg. *Calosphace*, our nuclear data suggest that sect. *Axillares* is sister to the *Hastatae* clade, but with strongly conflicting support (ASTRAL LPP = 0.38/ASTRAL BS = 86/concatenated BS = 92; 20/60 informative gene trees), while our plastid data place sect. *Axillares* as sister to all other *Calosphace* (BS = 100). There is also uncertainty about the deepest divergences in subg. *Salvia*, with weak support based on the ASTRAL and concatenated analyses.

**FIGURE 2:**
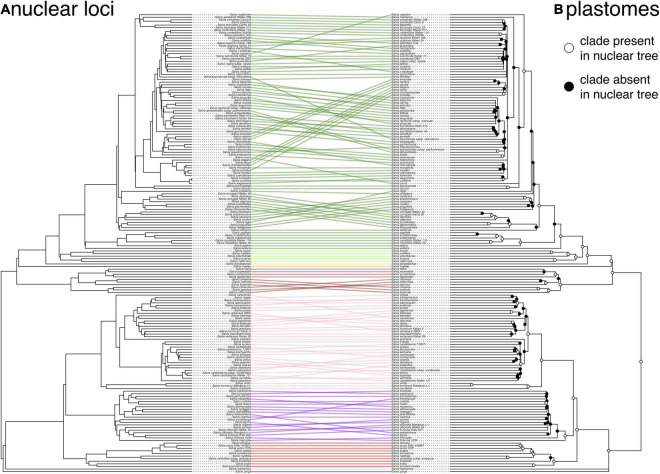
Tanglegram illustrating the cytonuclear discordance in *Salvia* based on the nuclear ASTRAL species tree **(A)** and the plastome tree **(B)**. Links connect identical tips, with nodes rotated to minimize the link overlap. Ingroup links are colored by subgenus. Clades that differ between the two trees are indicated by filled circles on the plastome tree. To minimize the discordance caused by the error in the gene tree estimation, clades in the plastome tree with <75% bootstrap support have been collapsed into polytomies.

Overall, there is fairly strong support (>90% support across all metrics) for many shallow-scale relationships, but support is notably very low or non-existent for some ASTRAL clades which do not appear in the best tree in the concatenated analysis or are in the low frequency in the BS replicates (Supplementary Figure S6), especially within the radiation of core *Calosphace* (e.g., relationships among *S. chamaedryoides* Cav., *S. coahuilensis* Fern., *S. microphylla* Kunth, and *S. muelleri* Epling), subg. *Salvia* (e.g., if *S. officinalis* s.s. is monophyletic or not), and subg. *Sclarea* (e.g., the placement of *S. sclarea* L.). There is a much more obvious infrageneric gene tree conflict between the nuclear loci and the plastome, with many shallower relationships conflicting between the two datasets, especially in subg. *Calosphace*, *Salvia*, and *Sclarea* (Figure 2).

Gene tree distances demonstrate significant gene tree conflicts within subgenera, both within and across genomic compartments (Table 1), and there are also significant differences in the gene tree distances among the subgenera for all three metrics (RF: *F*_5_,_454_ = 145.7, *p* < 0.0001; Nye: *F*_5_,_454_ = 98.6, *p* < 0.0001; CI: *F*_5_,_454_ = 92.4, *p* < 0.0001). *Post hoc* testing suggests that compared with the ASTRAL species tree, subg. *Glutinaria* and *Heterosphace* have significantly less discordant nuclear gene trees on average than all other subgenera, while subg. *Salvia* is more discordant than *Calosphace* for the Nye and CI metrics, and subg. *Sclarea* is more discordant than *Calosphace* for CI alone (Table 1). Likewise, although they are only point estimates, the discordance between the plastome and the ASTRAL tree is elevated for subg. *Sclarea* and especially, *Salvia* relative to all other subgenera (Table 1).

**TABLE 1:** Summary of the mean gene tree distances in *Salvia* and the selected subgenera within and across genomes.

clade	*n-*tips		nuclear loci			plastome	
		RF	Nye	CI	RF	Nye	CI
*Salvia*-wide	179	0.61^b^	0.41^b^	0.36^b^	0.63	0.37	0.29
subg. *Calosphace*	93	0.69^c^	0.52^d^	0.57^d^	0.72	0.41	0.45
subg. *Glutinaria*	10	0.29^a^	0.20^a^	0.24^a^	0.38	0.23	0.25
subg. *Heterosphace*	10	0.28^a^	0.18^a^	0.22^a^	0.08	0.08	0.07
subg. *Salvia*	37	0.67^bc^	0.44^bc^	0.50^c^	0.93	0.64	0.74
subg. *Sclarea*	20	0.70^c^	0.48^cd^	0.49^c^	0.78	0.46	0.51

*The three different distance metrics, Robinson–Foulds (RF), Nye, and clustering information (CI), compare the topology of the gene trees to the ASTRAL species tree, and we considered the branching order alone. Metrics were calculated on the gene trees that can be rooted and have clade occupancy > 75% of sampled tips. The distances were normalized so that they ranged from 0 to 1, with 0 indicating complete agreement between a gene tree and a species tree. The letters denote significantly different among-group differences for each metric in mean nuclear gene tree discordance compared to the species tree based on an ANOVA with post hoc testing at α = 0.05. Differences among the plastomes were not tested because they represent one gene tree.*

Compared with the expectation under ILS alone, the nuclear gene tree discordance in the observed gene trees is generally on par with or is less than what would be expected for under ILS based on RF distance but is greater than what would be expected in subg. *Calosphace*, *Salvia*, and *Sclarea* based on both the Nye and CI metrics (Table 2).

**TABLE 2:** Nuclear gene tree distances in *Salvia* and selected subgenera.

Clade	RF	Nye	CI	*t*_RF_	*p*_RF_	*t*_NYE_	*p*_NYE_	*t*_CI_	*p*_CI_
	mean (SD)	mean (SD)	Mean (SD)						
***Salvia*-wide**								
Observed	0.61 (0.07)	0.41 (0.06)	0.36 (0.06)	**–4.87**	**7.82 × 10^–6^**	**7.84**	**7.45 × 10^–11^**	**5.08**	**3.65 × 10^–6^**
Expected	0.65 (0.03)	0.35 (0.02)	0.32 (0.02)						
**subg. *Calosphace***									
Observed	0.69 (0.08)	0.52 (0.10)	0.57 (0.11)	**–2.92**	**4.39 × 10^–3^**	**10.67**	** < 1.00 × 10^–15^**	**8.83**	**1.12 × 10^–13^**
Expected	0.72 (0.05)	0.40 (0.03)	0.46 (0.04)						
**subg. *Glutinaria***									
Observed	0.29 (0.21)	0.20 (0.17)	0.24 (0.18)	–1.04	0.30	0.49	0.63	–0.029	0.98
Expected	0.32 (0.17)	0.19 (0.09)	0.24 (0.11)						
**subg. *Heterosphace***									
Observed	0.28 (0.17)	0.18 (0.11)	0.22 (0.13)	–1.06	0.29	0.98	0.33	–0.11	0.91
Expected	0.30 (0.17)	0.17 (0.09)	0.22 (0.11)						
**subg. *Salvia***									
Observed	0.67 (0.17)	0.44 (0.16)	0.50 (0.16)	–0.91	0.36	**4.70**	**8.84 × 10^–6^**	**2.56**	**0.012**
Expected	0.69 (0.12)	0.36 (0.07)	0.46 (0.09)						
**subg. *Sclarea***									
Observed	0.70 (0.10)	0.48 (0.10)	0.49 (0.11)	**–3.10**	**2.62 × 10^–3^**	**8.35**	**1.14 × 10^–12^**	**4.37**	**3.51 × 10^–5^**
Expected	0.74 (0.07)	0.38 (0.04)	0.43 (0.05)						

*The three different distance metrics, RF, Nye, and CI, compare the topology of the gene trees to the ASTRAL species tree, and we considered the branching order alone. The gene trees are either empirical trees that can be rooted and have clade occupancy > 75% of sampled tips (observed) or 1,000 gene trees simulated under the multispecies coalescent using the ASTRAL species tree (expected). The distances were normalized so that they ranged from 0 to 1, with 0 indicating complete agreement between a gene tree and the species tree. The t-values and associated p-values for each distance metric/clade combination are based on Welch’s t-test with the hypothesis that the mean tree distance for the observed and expected gene trees are equal, or in other words, that the tree distances based on empirical data are what would be expected under incomplete lineage sorting alone. Significant t/p-values at α = 0.05 are indicated in bold.*

### Phylogenetic Networks

**FIGURE 3:**
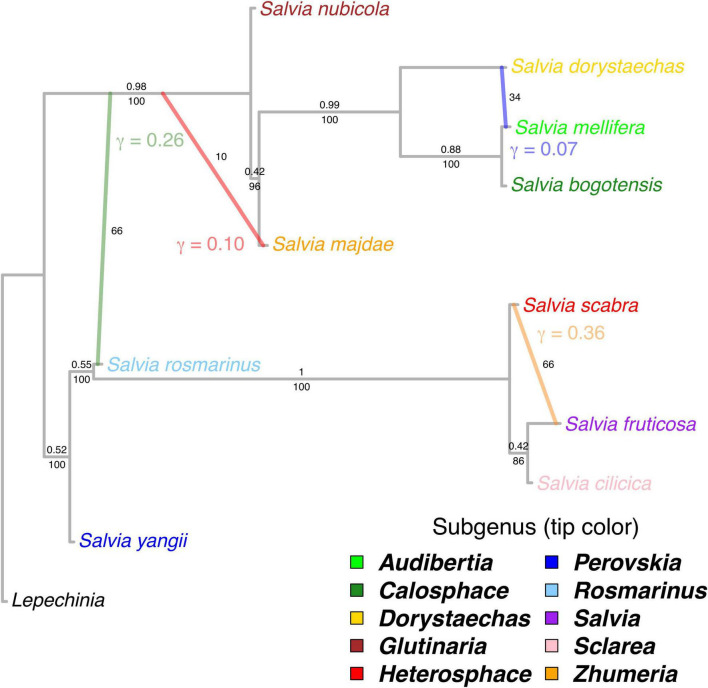
Phylogenetic network based on 57 nuclear genes depicting backbone relationships in *Salvia* with one exemplar per subgenus, with internal branch lengths in coalescent units. The best-fitting network has four reticulation events. Inheritance probabilities (γ) are indicated next to the lineage which is inferred to have received genetic material. The numbers above the branches on the bifurcating major topology are quartet concordance factors or the proportion of the genome supporting each quartet. The numbers below the major topology branches and to the right of hybrid branches are bootstrap support values. Ingroup tips are colored by subgenus.

The best phylogenetic network contained four reticulation events along the backbone of *Salvia* (hmax = 4). The major topology (i.e., bifurcating backbone) was identical to that recovered by the ASTRAL and concatenated analysis of all nuclear loci (Figure 3). Quartet CF along the backbone were generally > 0.50, and these edges received full BS support with the exception of the placement of subg. *Zhumeria* (CF = 0.42, BS = 96) and the sister relationship of subg. *Salvia* and *Sclarea* (CF = 0.42, BS = 86). While the BS analysis found evidence for horizontal gene flow, there was considerable uncertainty regarding the number and placement of reticulation events, with BS replicates recovering either three (52%) or four reticulation events. Inheritance probabilities (γ, the fraction of the nuclear genome involved in a reticulation event) for the four reticulation events on the best-fitting network ranged from 7 to 36% (Figure 3). The best network recovered gene flow from the stem of subg. *Salvia* to *Heterosphace* (γ = 0.36, BS = 66), stem subg. *Rosmarinus* to stem MRCA of *Calosphace* + *Glutinaria* (γ = 0.26, BS = 66), stem MRCA of subg. *Calosphace* + *Glutinaria* to *Zhumeria* (γ = 0.10, BS = 10), and stem subg. *Dorystaechas* to *Audibertia* (γ = 0.07, BS = 34). Alternative reticulation events found in frequency > 10% in the BS replicates involved the stem of subg. *Salvia* and *Sclarea* (BS = 22, with alternative relationships among *Heterosphace*, *Salvia*, and *Sclarea*), stem MRCA subg. *Rosmarinus* + *Salvia* and MRCA *Calosphace* + *Glutinaria* (BS = 28), stem subg. *Glutinaria* and stem *Audibertia* + *Calosphace* (BS = 40), and stem subg. *Glutinaria* and stem MRCA *Calosphace* + *Dorystaechas* (BS = 40).

## Discussion

### A Robust Phylogenetic Hypothesis for *Salvia*

Our results demonstrate that despite the gene tree discordance, the backbone relationships of *Salvia* are identical using nuclear and plastid data. These genomes support the monophyly of all currently recognized subgenera and are largely concordant regarding the intersubgeneric relationships, where supported. Discordance among nuclear loci can largely be reconciled by invoking ILS and horizontal gene flow.

Our results largely corroborate our previous analysis involving a larger number of loci with fewer terminals (Kriebel et al., 2019). Despite the fewer loci examined in this study, the average locus length is nearly twice as long (623 vs. 1,133 bp), presumably increasing the accuracy in the gene tree estimation. Additionally, compared with Kriebel et al. (2019) we found increased ASTRAL BS support for the monophyly of subg. *Audibertia* (>0.99 vs. 0.79) and for the sister relationship of subg. *Salvia* and *Sclarea* (0.99 vs. 0.79). However, we did not find convincing support for subg. *Audibertia* based on the ASTRAL LPP, and the monophyly for this subgenus based on the nuclear data has been somewhat unclear (Walker et al., 2004, 2015; Walker and Sytsma, 2007; Drew et al., 2017; Will and Claßen-Bockhoff, 2017), although it is clearly monophyletic based on the datasets with a good sampling of plastid loci (Walker et al., 2015; Supplementary Figure S5).

Unexpectedly, we found some uncertainty regarding the placements of subg. *Perovskia*, *Rosmarinus*, and *Zhumeria*, which were previously placed with BS = 100 in Kriebel et al. (2019). Nevertheless, the placement of all these subgenera, especially *Perovskia* and *Rosmarinus*, has varied widely across previous molecular studies incorporating low-copy nuclear loci (Drew et al., 2017), transcriptomes (Mint Evolutionary Genomics Consortium, 2018), nuclear ribosomal ITS/ETS (Drew and Sytsma, 2012; Will and Claßen-Bockhoff, 2017; Kriebel et al., 2019), and plastid data (Walker and Sytsma, 2007; Drew and Sytsma, 2012; Drew et al., 2017; Will and Claßen-Bockhoff, 2017; Zhao et al., 2020, 2021). The varying placement of subg. *Rosmarinus* across studies–and indeed across the loci examined in this study–can be explained by a combination of ILS and ancient horizontal gene flow (see below). This may be true for subg. *Zhumeria* as well, although it is less likely that horizontal gene flow has been involved in that case given the low BS support for any such gene flow.

While the backbone topology of the plastome of *Salvia* does not contradict that of the nuclear tree, it is still incompletely supported. However, it is unclear how much more information about the major relationships in *Salvia*, especially the relationships of subg. *Perovskia* and *Rosmarinus*, can be garnered from it. Analyses incorporating complete or nearly complete plastomes have failed to recover a fully supported backbone (Zhao et al., 2020; Supplementary Figure S5). Therefore, adding the portions of the plastome that we excluded does not seem to provide a viable solution. Mitogenomes, possibly combined with plastomes, are a possible avenue of research for a fully supported organellar phylogeny.

A final open question regarding the deeper-level phylogeny of *Salvia* is the relationship of the unsampled subg. *Meriandra*. We expect this subgenus to be closely related to subg. *Dorystaechas* and possibly even sister to it based on previous molecular results (Walker and Sytsma, 2007; Drew and Sytsma, 2012; Will and Claßen-Bockhoff, 2017; Kriebel et al., 2019). The placement of subg. *Meriandra* has important implications, not only for the historical biogeography of the genus but also implications for the timing and geographic location of any gene flow between the ancestors of subg. *Audibertia* and *Dorystaechas*, if present (see below).

### Evidence for Gene Flow in the Backbone of *Salvia*: But Where?

While the major topology of our phylogenetic network in *Salvia* is clear and strongly recovers the same bifurcating backbone found across other analyses, each with different assumptions, it also suggests that such a relatively simple tree may not be the best model of the phylogenetic history of *Salvia* (Figure 3). While all of our BS trees recovered at least three gene flow events, there is considerable uncertainty regarding which clades were involved in some of the horizontal gene flow events. We are, however, fairly certain that one gene flow event involved the stem MRCA of subg. *Glutinaria* and *Calosphace*, with the gene flow involving either the ancestor of subg. *Rosmarinus* alone (BS = 66) or the MRCA of subg. *Rosmarinus* and *Salvia* (BS = 28), and this likely explains the uncertainty regarding the placement of subg. *Perovskia* and *Rosmarinus*.

Likewise, it seems probable that the uncertainty regarding the branching order of subg. *Heterosphace*, *Salvia*, and *Sclarea* is the result of the gene flow. Although our phylogenetic network favors horizontal gene flow between subg. *Heterosphace* and *Salvia* as a better explanation for the discordance (BS = 66), an alternative resolution of the relationships with the gene flow between subg. *Salvia* and *Sclarea* is also possible (BS = 22). Given this result, it is also possible that the constraint of the level-1 network is not an appropriate model, and subg. *Salvia* may be a hybrid between subg. *Heterosphace* and *Sclarea*. This hypothesis requires further testing.

On the other hand, we found poor support for the remaining two inferred horizontal gene flow events on our best network, especially for the gene flow involving subg. *Zhumeria*. While slightly better supported, the horizontal gene flow between subg. *Audibertia* and *Dorystaechas* seem implausible since it necessitates the gene flow between their ancestors in North America and Southwest Asia (Kriebel et al., 2019). Despite its absence from the best network, the BS replicates suggest a strong possibility of gene flow involving subg. *Glutinaria*, especially with the MRCA of *Audibertia* + *Calosphace* (possibly extended to *Dorystaechas*), which would be more consistent with our current understanding of the historical biogeography of *Salvia*.

Apart from a level-1 network possibly being an unreasonable restriction to our dataset, the uncertainty in the placement of horizontal gene flow may be due to the relatively few loci employed here. For example, SNAq may recover false positive hybridization events in the datasets with < 100 loci (Solís-Lemus and Ané, 2016). Overall, our results highlight that it is essential to complement searches for best-fitting networks with BS analyses.

### Support for Key Infrageneric Structure

Our AHE data provides good support for many shallow-scale relationships in *Salvia* (Figure 1 and Supplementary Figures S1–S3). We clarify the placement of sect. *Axillares* within subg. *Calosphace*, with the nuclear evidence slightly favoring a hypothesis of the sister relationship of sect. *Axillares* and the *Hastatae* clade (sects. *Blakea*, *Hastatae*, and *Standleyana*), although the support based on ASTRAL LPP is noticeably weak. This relationship is identical to that suggested by the nuclear ribosomal DNA (Fragoso-Martínez et al., 2018; Kriebel et al., 2019, Appendix S7), but not the plastid data (Drew et al., 2017; Will and Claßen-Bockhoff, 2017; Fragoso-Martínez et al., 2018) or one low copy nuclear marker (*PPR*: Drew et al., 2017), which instead placed sect. *Axillares* as sister to the remainder of subg. *Calosphace*. It is still unclear if the uncertainty in the placement is due to ILS or gene flow, which should be investigated in future studies focused on subg. *Calosphace*, especially since the placement of sect. *Axillares* has important macroevolutionary implications.

Where our sampling of the Old World lineages permits, we corroborated relationships among deep subgeneric splits which were strongly supported in previous studies (subg. *Glutinaria*: Hu et al., 2018, subg. *Heterosphace*: Will and Claßen-Bockhoff, 2014, 2017). Within the other Old World subgenera, the support for relationships in previous studies has not been robust enough to warrant a discussion of the major relationships (Will and Claßen-Bockhoff, 2014, 2017), and thus our results, where it is well supported, are novel.

From a taxonomic perspective, it is encouraging that for the few species for which we have multiple accessions, morphologically defined species are monophyletic, paraphyletic due to the inclusion of only one other species, or essentially form a polytomy with other morphologically similar species, rather than polyphyletic and/or found in large polytomies (Figure 1 and Supplementary Figures S1–S3). This suggests that our AHE dataset has the power to not only resolve deeper relationships in *Salvia* but also to provide information pertinent to species delimitation.

### Infrageneric Structure: Incomplete Lineage Sorting and Horizontal Gene Flow Explain Strong Gene Tree Discordance

While phylogenomic datasets show great promise to resolve relationships in previously intractable angiosperm lineages, the irony is that many of these groups have undergone rapid radiations (Larson et al., 2020; Shee et al., 2020; Rose et al., 2021; Thomas et al., 2021), which increases the chance of gene tree heterogeneity due to ILS (Pamilo and Nei, 1988; Maddison, 1997; Oliver, 2013). Rampant gene tree discordance need not mean that species trees are poorly supported, provided that the discordance is consistent with the underlying model used to generate the species tree. Indeed, our analysis suggests that much of the gene tree discordance is at least consistent with ILS, given the high support for many infrageneric relationships based on our ASTRAL analysis (Figure 1 and Supplementary Figures S1, S2). Conversely, the low support for relationships in approaches that only take ILS into account may be due to either the lack of information about a given relationship in the underlying sequence data, or a more complex model of relationships (i.e., one involving horizontal gene flow). Our results demonstrate that while the average discordance of nuclear gene trees is consistent with what would be expected under ILS alone in the relatively under-sampled subg. *Glutinaria* and *Heterosphace*, it exceeds what would be expected under ILS in subg. *Calosphace*, *Salvia*, and *Sclarea* (Table 2). More strikingly, since that under ILS gene tree discordance should increase simply as a function of taxon sampling, it is notable that subg. *Salvia* and *Sclarea* have observed mean nuclear gene tree discordance on par with or slightly lower than that for subg. *Calosphace*, despite being represented by many fewer tips (Table 1).

One possible explanation for this is that the increased ILS results from the very rapid radiations of these clades, in combination with much younger crown ages for the MRCAs of what this study samples in subg. *Sclarea* (13.4 My) and *Salvia* (<7.8 My) relative to *Calosphace* (20.1 My) (Kriebel et al., 2019). However, based on the excess of the nuclear gene tree discordance in the aforementioned clades relative to the expectation under the multispecies coalescent, we suggest that in these clades, especially in subg. *Salvia* and *Sclarea*, the multispecies coalescent does not provide an ideal model of phylogenetic relationships. Instead, a model with horizontal gene flow in these lineages is likely a better explanation for the excess of gene tree discordance observed in our data. While another possibility for this pattern is that error in the gene tree estimation adds artificial discordance, we reject this as a major complicating factor given that we collapsed very poorly supported edges in observed gene trees.

The stark discordance present between the ASTRAL tree and the plastome tree at many shallow nodes in subg. *Calosphace*, *Salvia*, and *Sclarea*, with especially large distances between the nuclear and plastid trees in subg. *Salvia*, is also highly suggestive of an important contribution from horizontal gene flow, either hybridization or introgression (Figure 2). However, depending on the amount of past backcrossing, a signal for past gene flow may be absent from the nuclear genome in some cases. While we did not test it here, we do not think ILS is a likely explanation for the intergenomic gene tree conflict given the results from other angiosperm systems (Folk et al., 2017; Morales-Briones et al., 2018; Lee-Yaw et al., 2019; Rose et al., 2021), although error in the species tree estimation is a possible explanation. In future studies, we expect that relatively under-sampled subgenera should show increasing levels of cytonuclear discordance as we increase species sampling, especially within the subg. *Glutinaria* (Hu et al., 2018). The potentially confounding effects of polyploidy and whole-genome duplications (WGD) were not evaluated here but are being investigated.

Finally, it is worth a brief note concerning why we found that the mean RF tree distance often conflicts with the other distance metrics by demonstrating that the mean discordance is either on par with expectations under the multispecies coalescent or observed gene trees are, in some cases, less discordant. Despite being a widely used metric, RF distance is probably too conservative in penalizing against relatively minor topological differences (Smith, 2020a), as the movement of a single tip may result in maximum tree-to-tree distances even though all other tips show the same branching pattern. Thus, collapsing poorly supported edges of observed gene trees into polytomies downplays discordance, while any minor topological differences in the fully-resolved expected gene trees are penalized.

## Conclusion

Our updated AHE dataset provides evidence for a well-supported backbone of *Salvia* and indicates that there is an emerging consensus of relationships in the genus that extends across genomic compartments. Past difficulty in inferring relationships has likely been caused by a combination of uninformative markers, ILS, and horizontal gene flow. To the latter point, while our dataset clearly shows evidence of horizontal gene flow at deep and shallow scales in *Salvia*, we are presently unable to confidently demonstrate how many ancient gene flow events occurred and where they are placed. This highlights the importance of assessing the support for the best-fitting phylogenetic network, rather than only presenting the best network.

Several issues still need clarification, especially in the placement of subg. *Meriandra* and in the monophyly of subg. *Audibertia*. We are confident that future analyses using this same or expanded set of loci, in concert with an evaluation of polyploidy and WGD processes, will resolve these issues. Additionally, targeted analyses of clades or further methodological advances will allow us to tease apart horizontal gene flow at shallower scales. Our phylogenetic hypothesis, as well as future, time-calibrated phylogenetic hypotheses of the entire genus *Salvia*, its constituent subgenera, and targeted clades, will provide an invaluable framework for which to conduct multiple comparative analyses in this fascinating genus.

## Data Availability Statement

The data presented in the study are deposited in the NCBI Sequence Read Archive (SRA) as BioProject PRJNA773953.

## Author Contributions

JR, RK, BD, and KS conceived and undertook the project. JR, RK, LK, AD, JG-G, FC, EL, AL, and BD assisted with the data collection. JR analyzed the data and led the writing with contributions from all authors. All the authors contributed to the article and approved the submitted version.

## Conflict of Interest

The authors declare that the research was conducted in the absence of any commercial or financial relationships that could be construed as a potential conflict of interest.

## Publisher’s Note

All claims expressed in this article are solely those of the authors and do not necessarily represent those of their affiliated organizations, or those of the publisher, the editors and the reviewers. Any product that may be evaluated in this article, or claim that may be made by its manufacturer, is not guaranteed or endorsed by the publisher.

## Footnotes

1
www.anchoredphylogeny.com

## Supplementary Material

Supplementary Figure S1 The ASTRAL species tree of *Salvia* and outgroups, with local posterior probabilities on branches. Ingroup branches are colored by subgenus.

Supplementary Figure S2 The ASTRAL species tree of *Salvia* and outgroups, with bootstrap support on branches. Ingroup branches are colored by subgenus.

Supplementary Figure S3 The RAxML maximum likelihood tree of *Salvia* and outgroups based on the concatenated nuclear matrix, with bootstrap support on branches. Ingroup branches are colored by subgenus.

Supplementary Figure S4 Phyparts summary of gene trees. Pies at major nodes summarize the percentage of various phylogenetic signals across 101 gene trees which can be rooted. The numbers at the left of the pies show the total number of gene trees in which the clade is found, followed by the total number of gene trees that conflict with that clade. The remainder of the gene trees, if any, do not provide information on that particular relationship. Ingroup branches are colored by subgenus.

Supplementary Figure S5 The RAxML maximum likelihood tree of *Salvia* and outgroups based on entire plastomes, with bootstrap support on branches. GenBank accessions are removed so that the plastome tree matches the nuclear tree in the tip composition. Ingroup branches are colored by subgenus.

Supplementary Figure S6 Tanglegram illustrating the disagreement between the ASTRAL **(A)** and concatenated maximum likelihood **(B)** species trees based on nuclear data. Links connect identical tips, with nodes rotated to minimize link overlap. Ingroup branches are colored by subgenus. Clades that differ between the two trees are indicated by filled circles on the concatenated maximum likelihood tree.
