# Supplementary material for: Sage Insights Into the Phylogeny of Salvia: Dealing With Sources of Discordance Within and Across Genomes
Source: Front Plant Sci. 2021 Nov 24;12:767478. doi: 10.3389/fpls.2021.767478 (PMC8652245; doi:10.3389/fpls.2021.767478)
Supplement: Supplementary Figure S2 — The ASTRAL species tree of Salvia and outgroups, with bootstrap support on branches. Ingroup branches are colored by subgenus. [file Image_2.PDF]

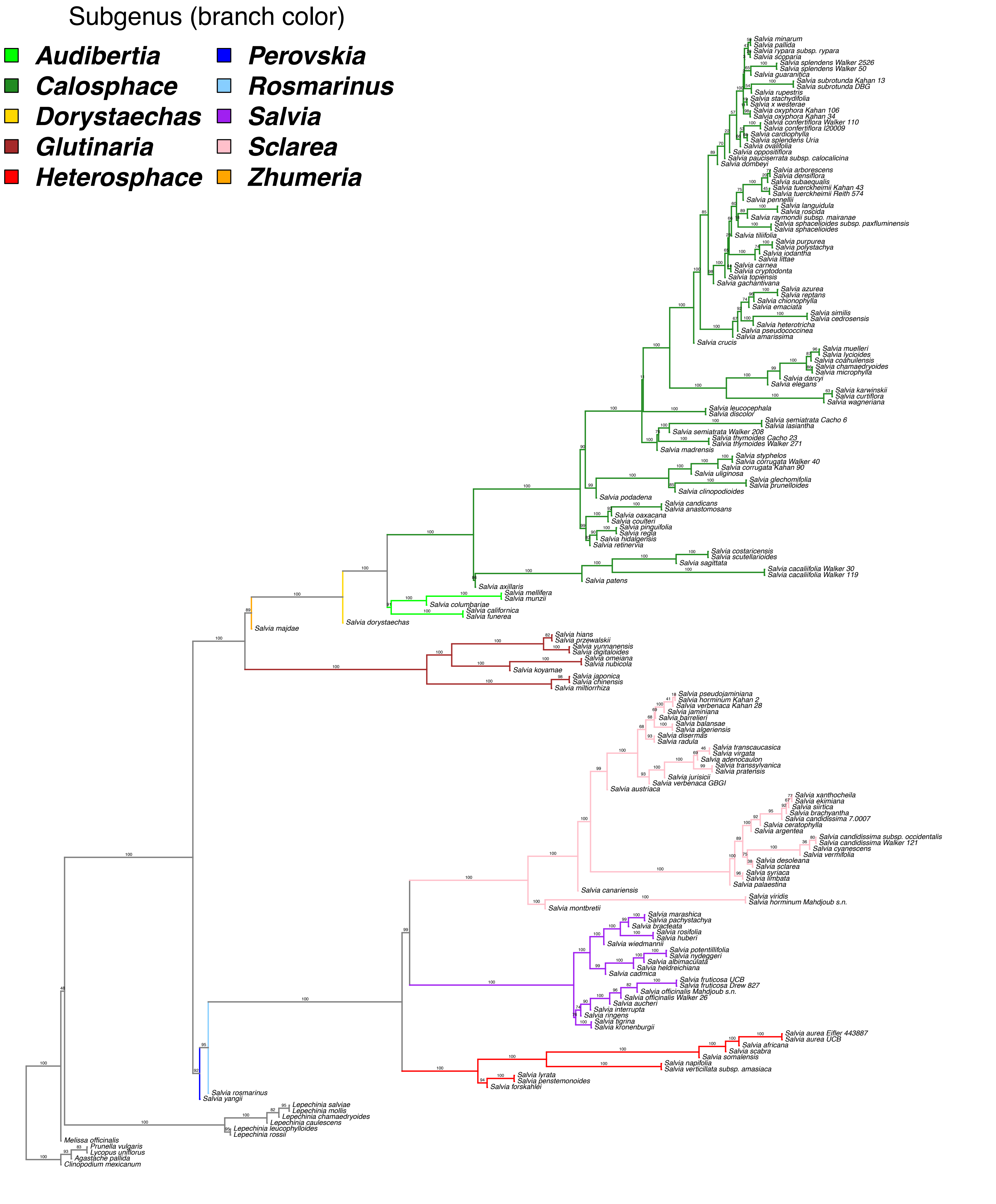

Subgenus (branch color)
Audibertia
Calosphace
Dorystaechas
Glutinaria
Heterosphace
Perovskia
Rosmarinus
Salvia
Sclarea
Zhumeria
Salvia minarum
Salvia pallida
Salvia rypara subsp. rypara
Salvia scoparia
Salvia splendens Walker 2526
Salvia splendens Walker 50
Salvia guaranitica
Salvia subrotunda Kahan 13
Salvia subrotunda DBG
Salvia rupestris
Salvia stachydifolia
Salvia x westerae
Salvia oxyphora Kahan 106
Salvia oxyphora Kahan 34
Salvia confertiflora Walker 110
Salvia confertiflora I20009
Salvia cardiophylla
Salvia splendens Uria
Salvia ovalifolia
Salvia oppositiflora
Salvia pauciserrata subsp. calocalicina
Salvia dombeyi
Salvia arborescens
Salvia densiflora
Salvia subaequalis
Salvia tuerckheimii Kahan 43
Salvia tuerckheimii Reith 574
Salvia pennellii
Salvia languidula
Salvia roscida
Salvia raymondii subsp. mairanae
Salvia sphacelioides subsp. paxfluminensis
Salvia sphacelioides
Salvia tiliifolia
Salvia purpurea
Salvia polystachya
Salvia iodantha
Salvia littae
Salvia carnea
Salvia cryptodonta
Salvia topiensis
Salvia gachantivana
Salvia azurea
Salvia reptans
Salvia chionophylla
Salvia emaciata
Salvia similis
Salvia cedrosensis
Salvia heterotricha
Salvia pseudococcinea
Salvia amarissima
Salvia crucis
Salvia muelleri
Salvia lycioides
Salvia coahuilensis
Salvia chamaedryoides
Salvia microphylla
Salvia darcyi
Salvia elegans
Salvia karwinskii
Salvia curtiflora
Salvia wagneriana
Salvia leucocephala
Salvia discolor
Salvia semiatrata Cacho 6
Salvia lasiantha
Salvia semiatrata Walker 208
Salvia thymoides Cacho 23
Salvia thymoides Walker 271
Salvia madrensis
Salvia styphelos
Salvia corrugata Walker 40
Salvia corrugata Kahan 90
Salvia uliginosa
Salvia glechomifolia
Salvia prunelloides
Salvia clinopodioides
Salvia podadena
Salvia candicans
Salvia anastomosans
Salvia oaxacana
Salvia coulteri
Salvia pinguifolia
Salvia regla
Salvia hidalgensis
Salvia retinervia
Salvia costaricensis
Salvia scutellarioides
Salvia sagittata
Salvia cacaliifolia Walker 30
Salvia cacaliifolia Walker 119
Salvia patens
Salvia axillaris
Salvia mellifera
Salvia munzii
Salvia columbariae
Salvia californica
Salvia funerea
Salvia dorystaechas
Salvia majdae
Salvia hians
Salvia przewalskii
Salvia yunnanensis
Salvia digitaloides
Salvia omeiana
Salvia nubicola
Salvia koyamae
Salvia japonica
Salvia chinensis
Salvia miltiorrhiza
Salvia pseudojaminiana
Salvia horminum Kahan 2
Salvia verbenaca Kahan 28
Salvia jaminiana
Salvia barrelieri
Salvia balansae
Salvia algeriensis
Salvia disermas
Salvia radula
Salvia transcaucasica
Salvia virgata
Salvia adenocaulon
Salvia transsylvanica
Salvia pratensis
Salvia jurisicii
Salvia verbenaca GBGI
Salvia austriaca
Salvia xanthocheila
Salvia ekimiana
Salvia siirtica
Salvia brachyantha
Salvia candidissima 7.0007
Salvia ceratophylla
Salvia argentea
Salvia candidissima subsp. occidentalis
Salvia candidissima Walker 121
Salvia cyanescens
Salvia vermifolia
Salvia desoleana
Salvia sclarea
Salvia syriaca
Salvia limbata
Salvia palaestina
Salvia canariensis
Salvia viridis
Salvia horminum Mahdjoub s.n.
Salvia montbretii
Salvia marashica
Salvia pachystachya
Salvia bracteata
Salvia rosifolia
Salvia huberi
Salvia wiedmannii
Salvia potentillifolia
Salvia nydeggeri
Salvia albimaculata
Salvia heldreichiana
Salvia cadmica
Salvia fruticosa UCB
Salvia fruticosa Drew 827
Salvia officinalis Mahdjoub s.n.
Salvia officinalis Walker 26
Salvia aucheri
Salvia interrupta
Salvia ringens
Salvia tigrina
Salvia kronenburgii
Salvia aurea Eifler 443887
Salvia aurea UCB
Salvia africana
Salvia scabra
Salvia somalensis
Salvia napifolia
Salvia verticillata subsp. amasiaca
Salvia lyrata
Salvia penstemonoides
Salvia forskahlei
Salvia rosmarinus
Salvia yangii
Lepechinia salviae
Lepechinia mollis
Lepechinia chamaedryoides
Lepechinia caulescens
Lepechinia leucophylloides
Lepechinia rossii
Melissa officinalis
Prunella vulgaris
Lycopus uniflorus
Agastache pallida
Clinopodium mexicanum
