# Supplementary material for: Sage Insights Into the Phylogeny of Salvia: Dealing With Sources of Discordance Within and Across Genomes
Source: Front Plant Sci. 2021 Nov 24;12:767478. doi: 10.3389/fpls.2021.767478 (PMC8652245; doi:10.3389/fpls.2021.767478)
Supplement: Supplementary Figure S3 — The RAxML maximum likelihood tree of Salvia and outgroups based on the concatenated nuclear matrix, with bootstrap support on branches. Ingroup branches are colored by subgenus. [file Image_3.PDF]

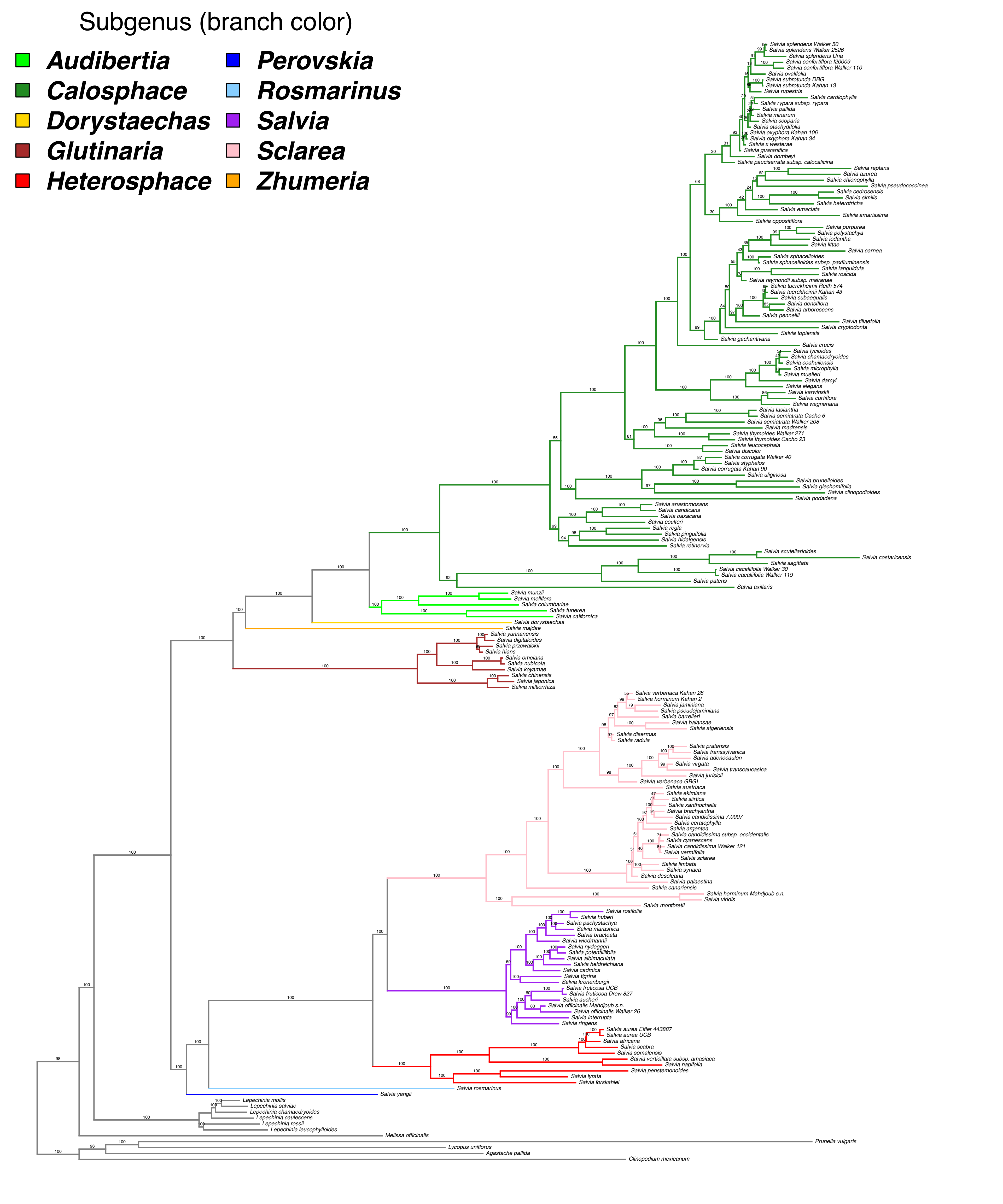
Subgenus (branch color)
Audibertia
Calosphace
Dorystaechas
Glutinaria
Heterosphace
Perovskia
Rosmarinus
Salvia
Sclarea
Zhumeria
Salvia splendens Walker 50
Salvia splendens Walker 2526
Salvia splendens Uria
Salvia confertiflora I20009
Salvia confertiflora Walker 110
Salvia ovalifolia
Salvia subrotunda DBG
Salvia subrotunda Kahan 13
Salvia rupestris
Salvia cardiophylla
Salvia rypara subsp. rypara
Salvia pallida
Salvia minarum
Salvia scoparia
Salvia stachydifolia
Salvia oxyphora Kahan 106
Salvia oxyphora Kahan 34
Salvia x westerae
Salvia guaranitica
Salvia dombeyi
Salvia pauciserrata subsp. calocalicina
Salvia reptans
Salvia azurea
Salvia chionophylla
Salvia pseudococcinea
Salvia cedrosensis
Salvia similis
Salvia heterotricha
Salvia emaciata
Salvia amarissima
Salvia oppositiflora
Salvia purpurea
Salvia polystachya
Salvia iodantha
Salvia littae
Salvia carnea
Salvia sphacelioides
Salvia sphacelioides subsp. paxfluminensis
Salvia languidula
Salvia roscida
Salvia raymondii subsp. mairanae
Salvia tuerckheimii Reith 574
Salvia tuerckheimii Kahan 43
Salvia subaequalis
Salvia densiflora
Salvia arborescens
Salvia pennellii
Salvia tiliaefolia
Salvia cryptodonta
Salvia topiensis
Salvia gachantivana
Salvia crucis
Salvia lycioides
Salvia chamaedryoides
Salvia coahuilensis
Salvia microphylla
Salvia muelleri
Salvia darcyi
Salvia elegans
Salvia karwinskii
Salvia curtiflora
Salvia wagneriana
Salvia lasiantha
Salvia semiatrata Cacho 6
Salvia semiatrata Walker 208
Salvia madrensis
Salvia thymoides Walker 271
Salvia thymoides Cacho 23
Salvia leucocephala
Salvia discolor
Salvia corrugata Walker 40
Salvia styphelos
Salvia corrugata Kahan 90
Salvia uliginosa
Salvia prunelloides
Salvia glechomifolia
Salvia clinopodioides
Salvia podadena
Salvia anastomosans
Salvia candicans
Salvia oaxacana
Salvia coulteri
Salvia regla
Salvia pinguifolia
Salvia hidalgensis
Salvia retinervia
Salvia scutellarioides
Salvia costaricensis
Salvia sagittata
Salvia cacaliifolia Walker 30
Salvia cacaliifolia Walker 119
Salvia patens
Salvia axillaris
Salvia munzii
Salvia mellifera
Salvia columbariae
Salvia funerea
Salvia californica
Salvia dorystaechas
Salvia majdae
Salvia yunnanensis
Salvia digitaloides
Salvia przewalskii
Salvia hians
Salvia omeiana
Salvia nubicola
Salvia koyamae
Salvia chinensis
Salvia japonica
Salvia miltiorrhiza
Salvia verbenaca Kahan 28
Salvia horminum Kahan 2
Salvia jaminiana
Salvia pseudojaminiana
Salvia barrelieri
Salvia balansae
Salvia algeriensis
Salvia disermas
Salvia radula
Salvia pratensis
Salvia transsylvanica
Salvia adenocaulon
Salvia virgata
Salvia transcaucasica
Salvia jurisicii
Salvia verbenaca GBGI
Salvia austriaca
Salvia ekimiana
Salvia siirtica
Salvia xanthocheila
Salvia brachyantha
Salvia candidissima 7.0007
Salvia ceratophylla
Salvia argentea
Salvia candidissima subsp. occidentalis
Salvia cyanescens
Salvia candidissima Walker 121
Salvia vermifolia
Salvia sclarea
Salvia limbata
Salvia syriaca
Salvia desoleana
Salvia palaestina
Salvia canariensis
Salvia horminum Mahdjoub s.n.
Salvia viridis
Salvia montbretii
Salvia rosifolia
Salvia huberi
Salvia pachystachya
Salvia marashica
Salvia bracteata
Salvia wiedmannii
Salvia nydeggeri
Salvia potentillifolia
Salvia albimaculata
Salvia heldreichiana
Salvia cadmica
Salvia tigrina
Salvia kronenburgii
Salvia fruticosa UCB
Salvia fruticosa Drew 827
Salvia aucheri
Salvia officinalis Mahdjoub s.n.
Salvia officinalis Walker 26
Salvia interrupta
Salvia ringens
Salvia aurea Eifler 443887
Salvia aurea UCB
Salvia africana
Salvia scabra
Salvia somalensis
Salvia verticillata subsp. amasiaca
Salvia napifolia
Salvia penstemonoides
Salvia lyrata
Salvia forskahlei
Salvia rosmarinus
Salvia yangii
Lepechinia mollis
Lepechinia salviae
Lepechinia chamaedryoides
Lepechinia caulescens
Lepechinia rossii
Lepechinia leucophylloides
Melissa officinalis
Prunella vulgaris
Lycopus uniflorus
Agastache pallida
Clinopodium mexicanum
