# Supplementary material for: Sage Insights Into the Phylogeny of Salvia: Dealing With Sources of Discordance Within and Across Genomes
Source: Front Plant Sci. 2021 Nov 24;12:767478. doi: 10.3389/fpls.2021.767478 (PMC8652245; doi:10.3389/fpls.2021.767478)
Supplement: Supplementary Figure S5 — The RAxML maximum likelihood tree of Salvia and outgroups based on entire plastomes, with bootstrap support on branches. GenBank accessions are removed so that the plastome tree matches the nuclear tree in the tip composition. Ingroup branches are colored by subgenus. [file Image_5.PDF]

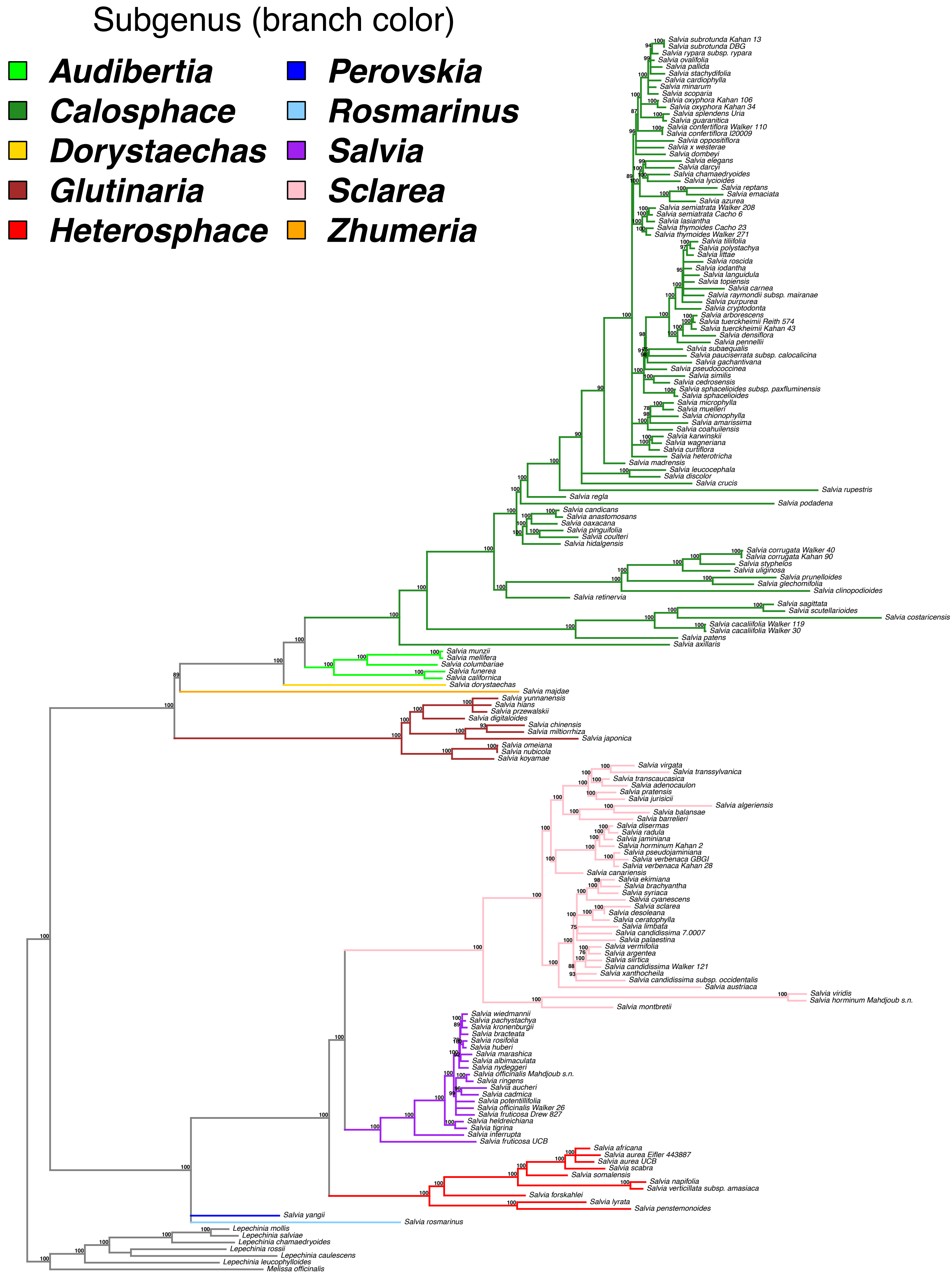

Subgenus (branch color)
Audibertia
Calosphace
Dorystaechas
Glutinaria
Heterosphace
Perovskia
Rosmarinus
Salvia
Sclarea
Zhumeria
Salvia subrotunda Kahan 13
Salvia subrotunda DBG
Salvia rypara subsp. rypara
Salvia ovalifolia
Salvia pallida
Salvia stachydifolia
Salvia cardiophylla
Salvia minarum
Salvia scoparia
Salvia oxyphora Kahan 106
Salvia oxyphora Kahan 34
Salvia splendens Uria
Salvia guaranitica
Salvia confertiflora Walker 110
Salvia confertiflora I20009
Salvia oppositiflora
Salvia x westerae
Salvia dombeyi
Salvia elegans
Salvia darcyi
Salvia chamaedryoides
Salvia lycioides
Salvia reptans
Salvia emaciata
Salvia azurea
Salvia semiatrata Walker 208
Salvia semiatrata Cacho 6
Salvia lasiantha
Salvia thymoides Cacho 23
Salvia thymoides Walker 271
Salvia tiliifolia
Salvia polystachya
Salvia littae
Salvia roscida
Salvia iodantha
Salvia languidula
Salvia topiensis
Salvia carnea
Salvia raymondii subsp. mairanae
Salvia purpurea
Salvia cryptodonta
Salvia arborescens
Salvia tuerckheimii Reith 574
Salvia tuerckheimii Kahan 43
Salvia densiflora
Salvia pennellii
Salvia subaequalis
Salvia pauciserrata subsp. calocalicina
Salvia gachantivana
Salvia pseudococcinea
Salvia similis
Salvia cedrosensis
Salvia sphacelioides subsp. paxfluminensis
Salvia sphacelioides
Salvia microphylla
Salvia muelleri
Salvia chionophylla
Salvia amarissima
Salvia coahuilensis
Salvia karwinskii
Salvia wagneriana
Salvia curtiflora
Salvia heterotricha
Salvia madrensis
Salvia leucocephala
Salvia discolor
Salvia crucis
Salvia rupestris
Salvia regla
Salvia podadena
Salvia candicans
Salvia anastomosans
Salvia oaxacana
Salvia pinguifolia
Salvia coulteri
Salvia hidalgensis
Salvia corrugata Walker 40
Salvia corrugata Kahan 90
Salvia styphelos
Salvia uliginosa
Salvia prunelloides
Salvia glechomifolia
Salvia clinopodioides
Salvia retinervia
Salvia sagittata
Salvia scutellarioides
Salvia costaricensis
Salvia cacaliifolia Walker 119
Salvia cacaliifolia Walker 30
Salvia patens
Salvia axillaris
Salvia munzii
Salvia mellifera
Salvia columbariae
Salvia funerea
Salvia californica
Salvia dorystaechas
Salvia majdae
Salvia yunnanensis
Salvia hians
Salvia przewalskii
Salvia digitaloides
Salvia chinensis
Salvia miltiorrhiza
Salvia japonica
Salvia omeiana
Salvia nubicola
Salvia koyamae
Salvia virgata
Salvia transsylvanica
Salvia transcaucasica
Salvia adenocaulon
Salvia pratensis
Salvia jurisicii
Salvia algeriensis
Salvia balansae
Salvia barrelieri
Salvia disermas
Salvia radula
Salvia jaminiana
Salvia horminum Kahan 2
Salvia pseudojaminiana
Salvia verbenaca GBGI
Salvia verbenaca Kahan 28
Salvia canariensis
Salvia ekimiana
Salvia brachyantha
Salvia syriaca
Salvia cyanescens
Salvia sclarea
Salvia desoleana
Salvia ceratophylla
Salvia limbata
Salvia candidissima 7.0007
Salvia palaestina
Salvia vermifolia
Salvia argentea
Salvia siirtica
Salvia candidissima Walker 121
Salvia xanthocheila
Salvia candidissima subsp. occidentalis
Salvia austriaca
Salvia viridis
Salvia horminum Mahdjoub s.n.
Salvia montbretii
Salvia wiedmannii
Salvia pachystachya
Salvia kronenburgii
Salvia bracteata
Salvia rosifolia
Salvia huberi
Salvia marashica
Salvia albimaculata
Salvia nydeggeri
Salvia officinalis Mahdjoub s.n.
Salvia ringens
Salvia aucheri
Salvia cadmica
Salvia potentillifolia
Salvia officinalis Walker 26
Salvia fruticosa Drew 827
Salvia heldreichiana
Salvia tigrina
Salvia interrupta
Salvia fruticosa UCB
Salvia africana
Salvia aurea Eifler 443887
Salvia aurea UCB
Salvia scabra
Salvia somalensis
Salvia napifolia
Salvia verticillata subsp. amasiaca
Salvia forskahlei
Salvia lyrata
Salvia penstemonoides
Salvia yangii
Salvia rosmarinus
Lepechinia mollis
Lepechinia salviae
Lepechinia chamaedryoides
Lepechinia rossii
Lepechinia caulescens
Lepechinia leucophylloides
Melissa officinalis
