# Supplementary figures and images for: Sage Insights Into the Phylogeny of Salvia: Dealing With Sources of Discordance Within and Across Genomes
Source: Front Plant Sci. 2021 Nov 24;12:767478. doi: 10.3389/fpls.2021.767478 (PMC8652245; doi:10.3389/fpls.2021.767478)

# (A) ASTRAL

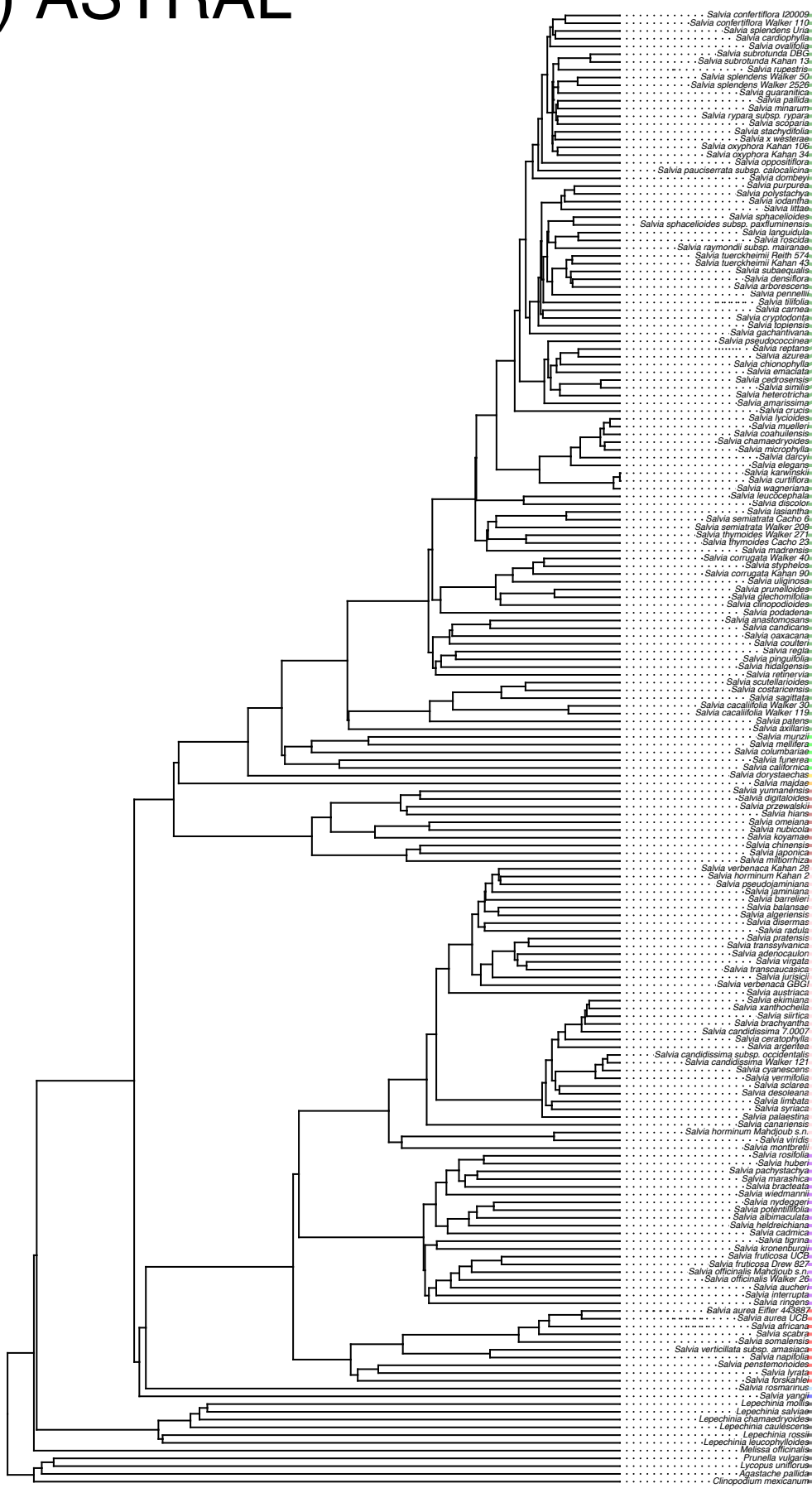

# (B) concatenated

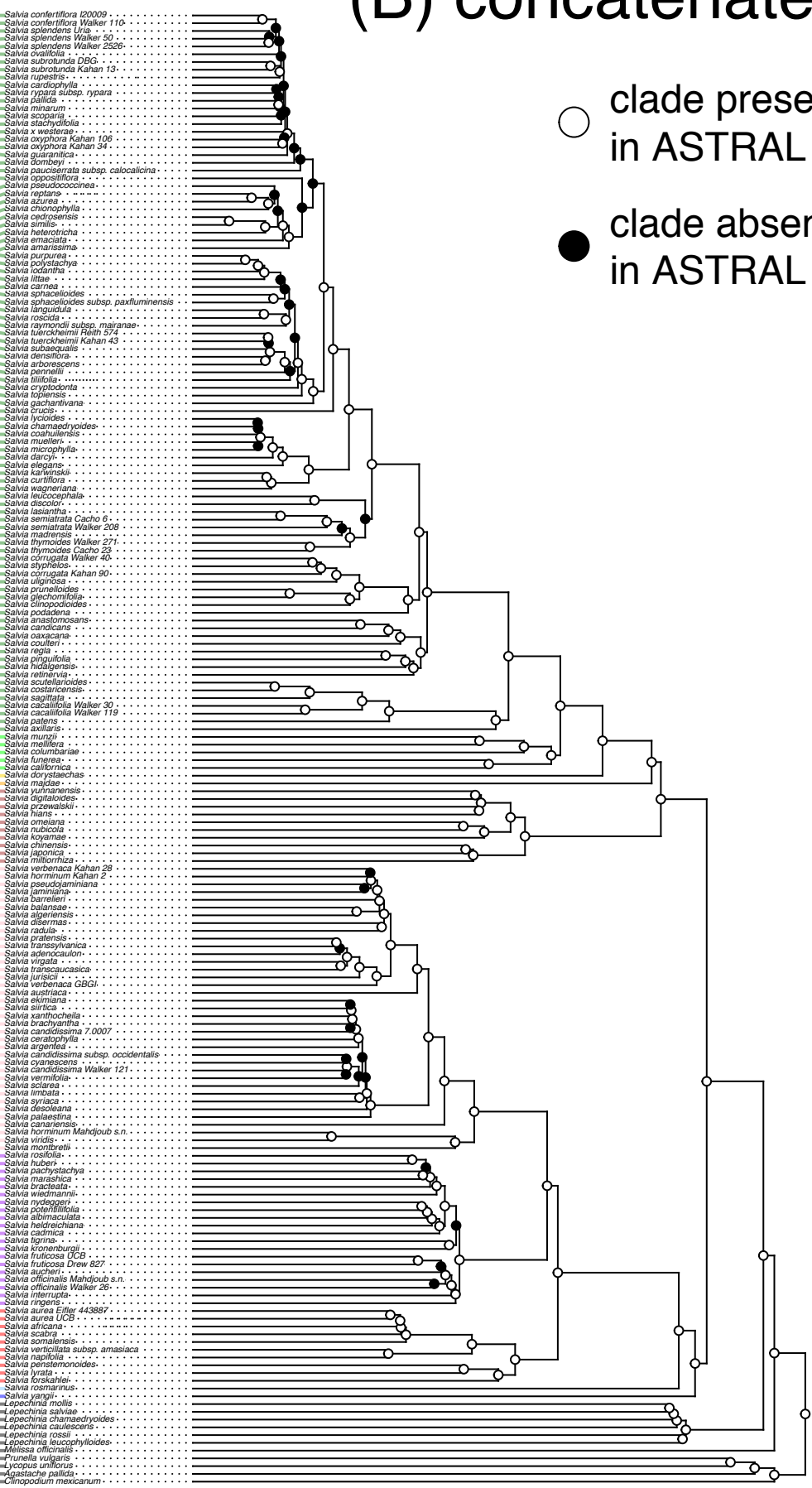

- clade present in ASTRAL tree
- clade absent in ASTRAL tree

Supplement: Supplementary Figure S6 — Tanglegram illustrating the disagreement between the ASTRAL (A) and concatenated maximum likelihood (B) species trees based on nuclear data. Links connect identical tips, with nodes rotated to minimize link overlap. Ingroup branches are colored by subgenus. Clades that differ between the two trees are indicated by filled circles on the concatenated maximum likelihood tree. [file Image_6.PDF]
